# Mitochondrial Epigenetic Changes Link to Increased Diabetes Risk and Early-Stage Prediabetes Indicator

**DOI:** 10.1155/2016/5290638

**Published:** 2016-05-19

**Authors:** Louise D. Zheng, Leah E. Linarelli, Joseph Brooke, Cayleen Smith, Sarah S. Wall, Mark H. Greenawald, Richard W. Seidel, Paul A. Estabrooks, Fabio A. Almeida, Zhiyong Cheng

**Affiliations:** ^1^Department of Human Nutrition, Foods, and Exercise, Fralin Translational Obesity Research Center, College of Agriculture and Life Science, Virginia Tech, Blacksburg, VA 24061, USA; ^2^Department of Family and Community Medicine, Carilion Clinic, Roanoke, VA 24016, USA; ^3^Department of Psychiatry, Carilion Clinic, Roanoke, VA 24014, USA; ^4^Department of Health Promotion, Social & Behavioral Health, College of Public Health, University of Nebraska Medical Center, Omaha, NE 68198, USA

## Abstract

Type 2 diabetes (T2D) is characterized by mitochondrial derangement and oxidative stress. With no known cure for T2D, it is critical to identify mitochondrial biomarkers for early diagnosis of prediabetes and disease prevention. Here we examined 87 participants on the diagnosis power of fasting glucose (FG) and hemoglobin A1c levels and investigated their interactions with mitochondrial DNA methylation. FG and A1c led to discordant diagnostic results irrespective of increased body mass index (BMI), underscoring the need of new biomarkers for prediabetes diagnosis. Mitochondrial DNA methylation levels were not correlated with late-stage (impaired FG or A1c) but significantly with early-stage (impaired insulin sensitivity) events. Quartiles of BMI suggested that mitochondrial DNA methylation increased drastically from Q1 (20 < BMI < 24.9, lean) to Q2 (30 < BMI < 34.9, obese), but marginally from Q2 to Q3 (35 < BMI < 39.9, severely obese) and from Q3 to Q4 (BMI > 40, morbidly obese). A significant change was also observed from Q1 to Q2 in HOMA insulin sensitivity but not in A1c or FG. Thus, mitochondrial epigenetic changes link to increased diabetes risk and the indicator of early-stage prediabetes. Further larger-scale studies to examine the potential of mitochondrial epigenetic marker in prediabetes diagnosis will be of critical importance for T2D prevention.

## 1. Introduction

Lifestyle contributes significantly to the epidemic of type 2 diabetes (T2D) [1–4]. The interactions between lifestyle factors and mitochondria (the primary metabolic platform) have been implicated in the pathogenesis of diabetes and obesity [5]. In particular, mitochondria undergo aberrant alteration and cause oxidative stress in diabetic subjects, and redox active compounds have shown promising potential to treat T2D [5–8]. Nevertheless, no known cure has been developed for diabetes to date [9, 10], and early diagnosis of prediabetes for intervention has been an important strategy to prevent T2D [3]. To this end, biomarkers that can signify progression or early stage of prediabetes will be of clinical significance.

The risk of T2D increases with weight gain. For instance, the lifetime risk of developing diabetes at an age of 18 years increases by 9.9%, 37.2%, and 50.5% in overweight (25 < BMI < 30), obese (30 < BMI < 35), and severely obese (>35) males, respectively, compared with normal weight individuals; for female subjects, it increases by 18.3%, 37.5%, and 57.3%, respectively [11]. As such, the American Diabetes Association (ADA) recommends BMI as a primary index to screen for high-risk population and recommend of further testing to assess risk of future diabetes development in asymptomatic people [12]. Following an initial weight screening, individuals having a fasting glucose (FG) ranging from 100 mg/dL (5.6 mmol/L) to 125 mg/dL (6.9 mmol/L) or an A1c of 5.7–6.4% are diagnosed as prediabetic [12]. Of note, the existing discordance between FG and A1c may lead to conflicting results, underscoring the utmost importance of identifying new markers that can consistently identify prediabetic individuals as early as possible for T2D prevention [13–15].

The effectiveness of diabetes prevention via lifestyle modification is strongly associated with improved insulin sensitivity [16] and epigenetic adaptation, including DNA methylation on the nuclear genome and mitochondrial genome [5, 17]. Recently, we identified an insulin signaling-epigenetic-genetic axis in the regulation of mitochondria, the major cellular metabolic platform that responds robustly to interventions [6, 18–23]. However, it is largely unknown whether mitochondrial epigenetic signature reflects prediabetes progression and how it interacts with the current diagnostic indices (A1c and FG). In the present work, we studied the interactions of mitochondrial DNA methylation with FG, A1c, and insulin sensitivity, the parameters that change stage-dependently during prediabetes progression [24–26]. DNA methylation in mitochondrial NADH dehydrogenase 6 (ND6) and displacement loop (D-loop) region was significantly correlated with changes in insulin sensitivity (the event starting in an earlier stage [24–26]) but had marginal interaction with A1c and FG (the later-stage markers in prediabetes progression [24–26]). Upon quartilizing BMI (the primary risk index for prediabetics screening [11, 12]), we found significant changes in mitochondrial epigenetics during the progression from Q1 (20 < BMI < 24.9, lean) to Q2 (30 < BMI < 34.9, obese), but not from Q2 to Q3 (35 < BMI < 39.9, severely obese) or Q3 to Q4 (BMI > 40, morbidly obese); by contrast, FG showed significant changes only from Q3 to Q4, and the levels of A1c were unchanged in all cases. Discordance was observed when FG or A1c was used to diagnose prediabetes, presumably because they indicate late but different stages of prediabetes. Together, our data suggest that mitochondrial epigenetic changes link to diabetes risk and signify early-stage prediabetes, targeting which may have the potential to achieve early diagnosis of prediabetes for T2D intervention.

## 2. Materials and Methods 

### 2.1. Ethics Statement

A written informed consent was obtained from each participant. All procedures were conducted in accordance with NIH Guidelines, and the research protocol was approved by Institutional Review Boards (IRB) at Carillion Clinic and at Virginia Tech.

### 2.2. Study Patients, Sample Inclusion, and Blood Chemistry

This study included 87 participants at baseline (before intervention), which were recruited for a larger diabetes prevention trial (diaBEAT-it trial) [27]. Twelve individuals were lean, and 75 participants were obese or overweight (Table 1). All participants completed an intake questionnaire about medical history, current medications, and current health behaviors. For blood collection, the participants were instructed to fast overnight (10–12 hours) before their scheduled blood draw at Solstas Labs facility (Roanoke, Virginia) [18]. Fasting venous blood samples were collected in EDTA tubes to study lipid profile (triglyceride, total cholesterol, HDL-cholesterol, and LDL-cholesterol), fasting plasma glucose, fasting plasma insulin, and HbA1c. The homeostasis model assessment for insulin resistance (HOMA-IR) index was calculated by (fasting insulin (*μ*IU/mL) × fasting glucose (mg/dL)/405) as previously described [18, 28, 29]. In addition, buffy coats (white blood cells) were prepared from fasting blood samples and stored at −80°C for later DNA extraction and methylation analysis [18, 30, 31].

### 2.3. DNA Isolation and Bisulfite Conversion

Fast extraction of high-quality DNA from buffy coats was conducted with QIAamp DNA Blood Mini Kits (Qiagen, Hilden, Germany), which utilize a silica-membrane-based DNA purification. DNA quality and quantity were determined on a Synergy H4 Hybrid Multi-Mode Microplate Reader (BioTek Instruments, Winooski, VT, USA). For bisulfite treatment and purification of converted DNA, we used EpiTect Bisulfite Kits (Qiagen). During bisulfite treatment, the methylated cytosines were conserved but unmethylated cytosines converted into uracils, and the purified DNA was used for methylation analysis [18].

### 2.4. Mitochondrial DNA Methylation

Methylation specific PCR (MSP) was employed to analyze mitochondrial DNA methylation in ND6 and D-loop region [18, 32, 33]. The bisulfite modified DNA was used as a template for MSP reactions on a ViiA*™* 7 Real-Time PCR System, using iQ*™* SYBR® Green Supermix (Bio-Rad Laboratories, Hercules, CA, USA). Two MSPs were performed simultaneously to detect the methylated and unmethylated DNA for each sample, and the percentage of methylated DNA was calculated as described previously [18, 32, 33]. The primers used in this study were TAGGAATTAAAGATAGATATTGCGA (forward) and 5′-ACTCTCCA TACATTTAATATTTTCGTC-3′ (reverse) for methylated D-loop; 5′-GGTAGGAATTAAA GATAGATATTGTGA-3′ (forward) and 5′-ACTCTCCATACATTTAATATTTTCATC-3′ (reverse) for unmethylated D-loop; 5′-TTTCGTATTAATAGGATTTTTTCGA-3′ (forward) and 5′-AATTATCTTTAAATATACTACAACGAT-3′ (reverse) for methylated ND6; 5′-TTTTGTATTAATAGGATTTTTTTGA-3′ (forward) and 5′-ATAATTATCTTTAAATAT ACTACAACAAT-3′ (reverse) for unmethylated ND6 [32]. Primer design was performed with the MethPrimer program, based on the sequence of human mitochondrial genome (>gi|251831106|ref|NC_012920.1|Homo sapiens mitochondrion, complete genome) [34].

### 2.5. Statistical Analysis

Pearson's correlation and regression analysis were applied to examine the relationships among obesity-related risk factor, metabolic indexes, and mitochondrial DNA methylation levels. In some cases, logarithm-transformed data were used for skewed variables (e.g., HOMA-IR). The data were expressed as the mean ± SE unless otherwise specified, and analysis of variance (ANOVA) was conducted to determine *p* values; *p* < 0.05 was considered statistically significant.

## 3. Results

### 3.1. Metabolic Changes with BMI

Compared with the lean (BMI = 22.7 ± 0.6) group, overweight and obese (BMI = 37.1 ± 1.8) individuals showed significant elevation in fasting glucose (83.4 versus 96.1 mg/dL, *p* < 0.01) and insulin (10.0 versus 24.6 *μ*IU, *p* < 0.05) levels (Table 1). In line with the notion that an increase in fasting insulin (FI) indicates impairment of insulin signaling [35, 36], we found that the homeostasis model assessment-estimated insulin resistance (HOMA-IR) index increased from 2.1 in the lean group to 6.0 in the overweight and obese group (*p* < 0.05) (Table 1). In addition, lipid profile was dysregulated in the overweight and obese individuals, showing significant elevation in LDL, VLDL, total cholesterol, and triglyceride (Table 1). However, A1c showed no difference between the two groups (5.5 versus 5.7, *p* > 0.05). These data suggest that BMI can reflect the changes in glucose and lipid metabolism, as well as the impairment in insulin signaling, supporting the notion that BMI represents an important risk index for both prediabetes and T2D development [11, 37, 38].

### 3.2. Diagnosis of Prediabetes Using A1c and FG

**Figure 1:**
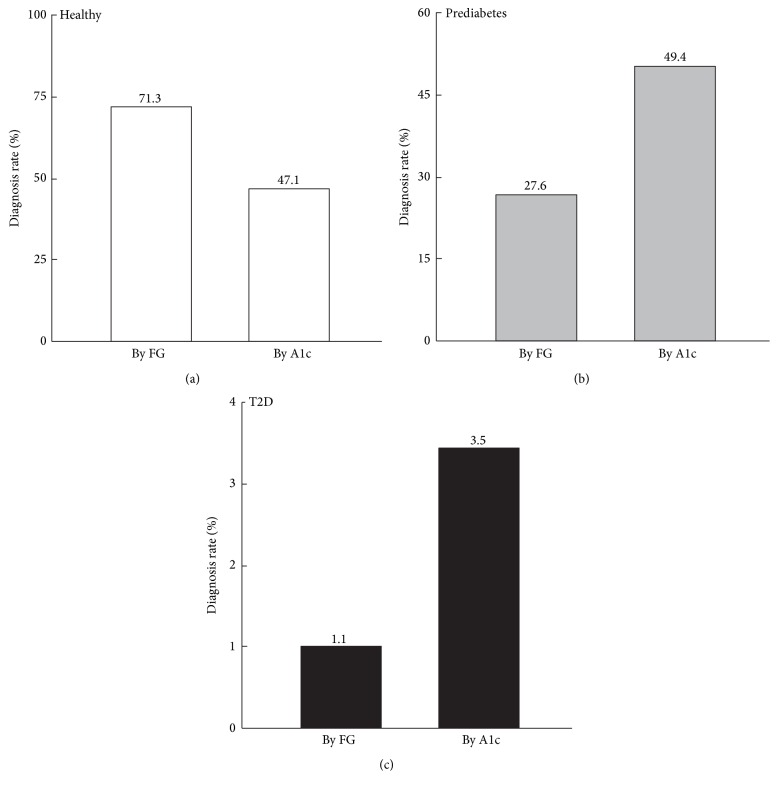
The diagnosis of healthy (a), prediabetes (b), and T2D (c) among 87 participants using the standards recommended by ADA for fasting glucose (FG) and hemoglobin A1c (A1c).

The observation of no difference in A1c between the low-risk (BMI = 22.7 ± 0.6) and high-risk (BMI = 37.1 ± 1.8) groups prompted us to examine the power of A1c in diagnosing prediabetes. According to the standards recommended by ADA [12], A1c indicated that 49.4% of the 87 participants had prediabetes (A1c = 5.7–6.4%), 3.5% had T2D (A1c ≥ 6.5%), and 47.1% were considered healthy (A1c = 4.8–5.6%). However, FG suggested that 71.3% of the participants were healthy, 27.6% were prediabetic, and only 1.1% had T2D (Figure 1). This discordance in A1c and FG recapitulates the findings in previous reports and underscores the urgent need of additional markers for reliable and consistent diagnosis [13–15].

### 3.3. Insulin Resistance Reflected Diabetes Risk Better than A1c and FG

**Figure 2:**
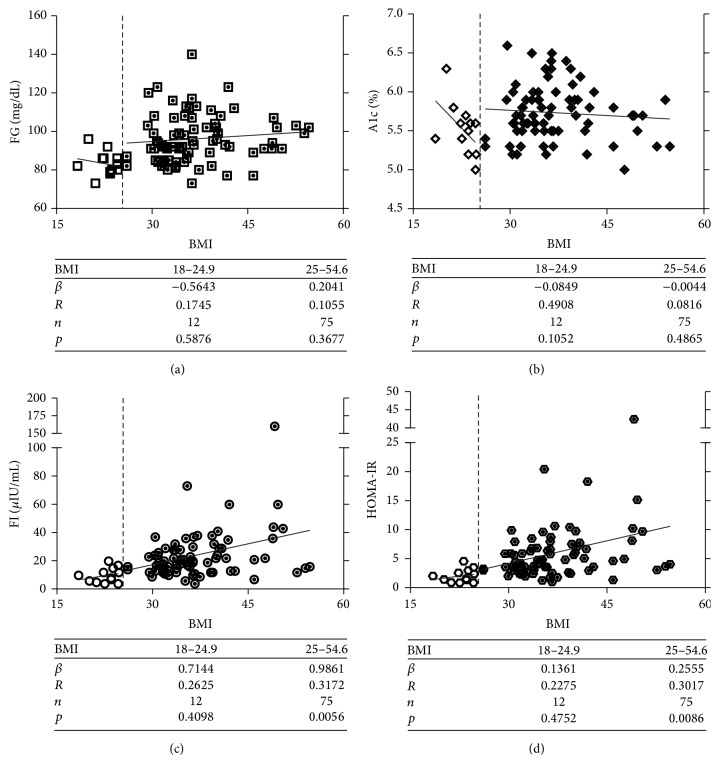
The correlations of obesity-associated diabetes risk index (BMI) with current diagnostic indices and insulin sensitivity. (a) FG showed no significant correlation with BMI. (b) A1c showed no significant correlation with BMI. (c-d) Decrease of insulin sensitivity was significantly associated with BMI in overweight and obese participants, as indicated by FI (c) and HOMA-IR (d).

Prediabetes is characterized by impaired fasting glucose and insulin sensitivity, which progress differently from A1c before the onset of T2D (Supplemental Figure  1 in Supplementary Material available online at http://dx.doi.org/10.1155/2016/5290638) [24–26]. Specifically, drastic changes were observed in insulin sensitivity 5 years prior to the diagnosis of T2D, which is 3 years earlier than FG and 4 years earlier than A1c (Supplemental Figure  1) [24–26]. In the lean or healthy group (BMI = 22.7 ± 0.6), regression analysis showed no significant correlation of BMI with FG (*p* = 0.5876), A1c (*p* = 0.1052), FI (*p* = 0.4095), or HOMA-IR (*p* = 0.4752) (Figure 2). Surprisingly, FG and A1c also failed to correlate with BMI in the high-risk (BMI = 37.1 ± 1.8) group (*p* = 0.3677 and 0.4865, resp., Figures 2(a) and 2(b)). By contrast, FI and HOMA-IR showed significant correlation with BMI (*p* = 0.0056 and 0.0086, resp., Figures 2(c) and 2(d)). Logarithm-transformed HOMA-IR values showed a similar correlation with BMI (Supplemental Figure  2). These data suggest that insulin resistance indices can better reflect diabetes risk. Given that impaired insulin sensitivity is observed prior to impaired FG and A1c in prediabetes [24–26], use of insulin resistance index may be superior to A1c or FG for early detection of prediabetes.

### 3.4. Mitochondrial Epigenetic Changes Were Associated with Insulin Resistance

**Figure 3:**
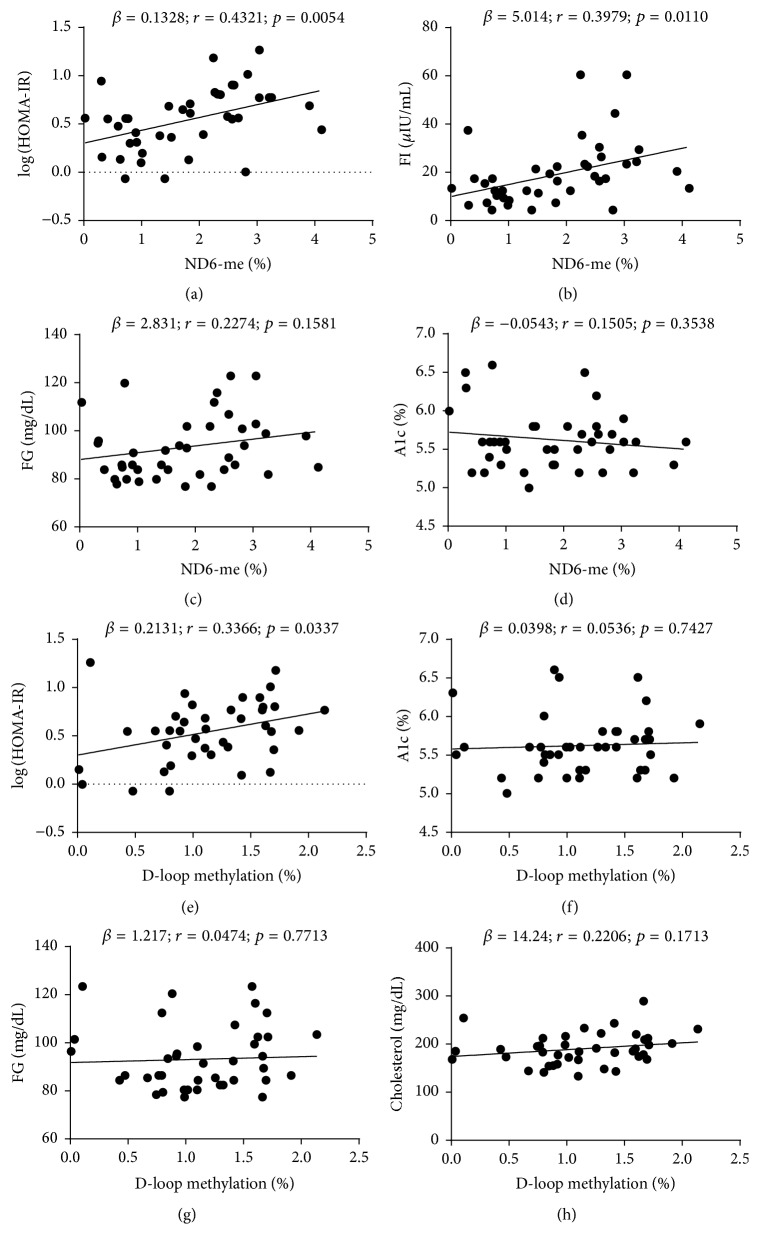
Correlation of mitochondrial DNA methylation with insulin sensitivity (HOMA-IR and FI) and current diagnostic indices (FG and A1c). (a-b) HOMA-IR and FI showed significant interaction with ND6 methylation; *n* = 40. (c) FG showed no correlation with ND6 methylation; *n* = 40. (d) A1c showed no correlation with ND6 methylation; *n* = 40. (e) HOMA-IR showed significant interaction with D-loop methylation; *n* = 40. (f) FG showed no correlation with D-loop methylation; *n* = 40. (g) A1c showed no correlation with D-loop methylation; *n* = 40. (h) Cholesterol showed no correlation with D-loop methylation; *n* = 40. More details about correlation of DNA methylation in mitochondrial D-loop with lipid profile can be found in Supplemental Figure  4.

The onset of T2D in prediabetic individuals can be effectively delayed or prevented by lifestyle intervention, in response to which mitochondria undergo epigenetic adaptation [1–3, 5, 17, 18, 39]. To examine how mitochondrial epigenetics interacts with A1c, FG, and insulin resistance, we collected additional blood samples from a subset (40 out of the 87, including 8 lean/healthy, and 32 obese) of participants and analyzed DNA methylation in mitochondrial ND6 and D-loop, the regions critical for mitochondrial function and DNA replication [18, 32]. The 40 individuals represented well the 87 participants, as demonstrated by the relations of BMI with A1c, FG, FI, and HOMA-IR (Supplemental Figure  3). More importantly, ND6 methylation level showed significant correlation with insulin resistance indices (*p* = 0.0054 for HOMA-IR and 0.0110 for FI; Figures 3(a) and 3(b)), but it had marginal interaction with FG (*p* = 0.1581; Figure 3(c)) and A1c (*p* = 0.3538; Figure 3(d)). Further regression analyses suggested that ND6 methylation had no significant correlation with lipid profile or age (Supplemental Figure  4, A-D, or data not shown), consistent with previous studies of global DNA methylation [40, 41]. This phenotype was recapitulated by D-loop methylation, which was significantly correlated with insulin resistance (HOMA-IR, *p* = 0.0337; Figure 3(e)), but not with A1c (*p* = 0.7427; Figure 3(f)) or FG (*p* = 0.7713; Figure 3(g)) or lipid profile (Figure 3(h), Supplemental Figure  4, E-H). These data suggest that mitochondrial epigenetic signature may represent an early-stage marker of prediabetes and its alteration parallels with the development of insulin resistance.

### 3.5. Mitochondrial Epigenetic Traits Were Associated with Increase Diabetes Risk and Signified Early-Stage Prediabetes

**Figure 4:**
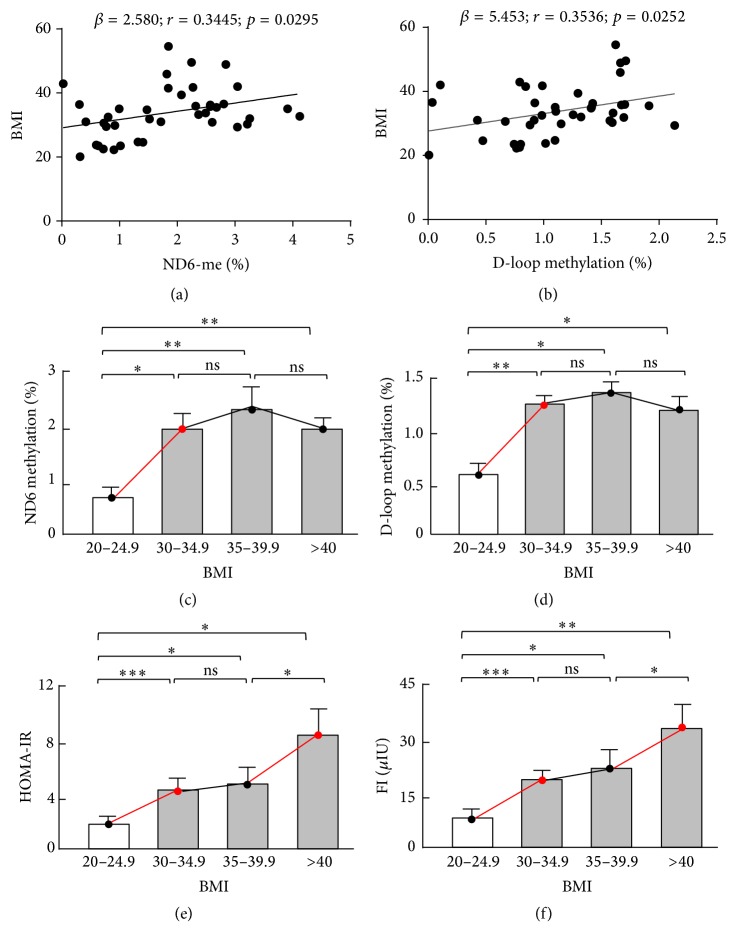
Changes in mitochondrial DNA methylation and insulin sensitivity versus different levels of diabetes risk factor (BMI) or different stages of disease progression. (a) ND6 methylation showed significant correlation with BMI; *n* = 40. (b) D-loop methylation showed significant correlation with BMI; *n* = 40. (c) ND6 methylation increased drastically from low-risk (20 < BMI < 24.9; *n* = 8) to high-risk (30 < BMI < 34.9; *n* = 15) stages, with no further significant changes during advanced progression (BMI > 35; *n* = 17). (d) D-loop methylation increased drastically from low-risk (20 < BMI < 24.9; *n* = 8) to high-risk (30 < BMI < 34.9; *n* = 15) stages, with no further significant changes during advanced progression (BMI > 35). (e) HOMA-IR increased drastically from low-risk (20 < BMI < 24.9; *n* = 8) to high-risk (30 < BMI < 34.9; *n* = 15) stages and underwent a second significant elevation during advanced progression (from 35 < BMI < 39.9 to BMI > 40; *n* = 9 and 8, resp.). (f) As an alternative index of insulin resistance, FI increased drastically from low-risk (20 < BMI < 24.9; *n* = 8) to high-risk (30 < BMI < 34.9; *n* = 15) stages and underwent a second significant elevation during advanced progression (from 35 < BMI < 39.9 to BMI > 40; *n* = 9 and 8, resp.). ^*∗*^ *p* < 0.05; ^*∗∗*^ *p* < 0.01; ^*∗∗∗*^ *p* < 0.001; ns: not significant. Red color indicates the dramatic changes in a parameter.

Weight gain is an established risk factor for prediabetes and T2D: an increase in BMI indicates a higher risk [11, 37, 38]. Intriguingly, DNA methylation levels of both ND6 and D-loop showed significant correlation with BMI (*p* = 0.0295 and 0.0252, resp., Figures 4(a) and 4(b)). To test how mitochondrial epigenetic changes progress with this risk factor, we plotted the epigenetics data with quartiles of BMI: 20–24.9 (Q1, lean or healthy), 30–34.9 (Q2, obese), 35–39.9 (Q3, severely obese), and >40 (Q4, morbidly obese). In comparison with Q1, the other three quartiles all showed significant increase in ND6 and D-loop methylation (Figures 4(c) and 4(d)). However, no further significant changes in DNA methylation were observed from Q2 to Q3 or to Q4. By contrast, insulin resistance indices (HOMA-IR and FI) underwent significant elevation from Q1 to Q2 and additionally from Q3 to Q4 (Figures 4(e) and 4(f)). The continuous changes in insulin resistance indices during high-risk stages (Q3 and Q4) are consistent with the trajectory showing that HOMA insulin sensitivity decreased steeply until the diagnosis of T2D (Supplemental Figure  1) [24, 25]. Of note, A1c showed marginal changes in all stages (from Q1 to Q4), and a significant increase in FG was observed only during advanced progression (Q3 and Q4) stages (Supplemental Figure  5). These results support the notion that FG is a late-stage marker of prediabetes progression, and change of A1c is an even later-stage event (Supplemental Figure  1) [24–26]. Importantly, we found that mitochondrial epigenetic changes were most sensitive when prediabetes progressed from low-risk (Q1) to high-risk (Q2) stages but less responsive to further progression in higher risk (Q3 and Q4) stages. Therefore, targeting mitochondrial epigenetic markers has the potential to identify early-stage prediabetes for as-early-as-possible lifestyle intervention.

## 4. Discussion

DNA methylation is a reversible epigenetic process that regulates genetic predisposition and gene expression and interacts with environmental cues [5, 40, 41]. The global DNA methylation and its interaction with age, metabolic markers, sex hormone, inflammation, and lifestyle behaviors in T2D and obese patients have been investigated in previous studies [40–45]. Earlier studies suggested that global DNA methylation was associated with glycemic and lipid profiles [42], but increasing evidence emerges and indicates no or opposite correlations with blood glucose or lipids [41, 43–45]. A 6-month exercise intervention was able to significantly reverse the altered DNA methylation in skeletal muscle from first-degree relatives of T2D patients and in adipose tissues from obese and T2D individuals [46, 47]. Upon measuring DNA methylation of long interspersed nucleotide element 1 (LINE-1) by pyrosequencing and global DNA methylation by the luminometric methylation assay (LUMA), Delgado-Cruzata et al. found that changes in weight, diets, or calorie intake affected global DNA methylation [45]. While the phenotype was recapitulated by an intervention study conducted by Martín-Núñez and colleagues [44], Duggan et al. suggested that 12-month lifestyle changes led to significant weight loss with no effects on global DNA methylation [43]. These studies have elegantly unraveled an epigenetic link to metabolic diseases, but the relationship between global DNA methylation and environmental and metabolic markers is more complex than expected and warrants further investigation.

Mitochondria underpin metabolic homeostasis [48–50]. We and others have shown that mitochondria undergo genetic, epigenetic, and functional changes in subjects with obesity, insulin resistance, or T2D [5, 18, 50–52]. In the present study, we investigated the relationships between mitochondrial DNA methylation and stage-dependent metabolic markers and diabetes risk index (BMI). It is known that the progression of prediabetes to T2D shows stage-dependent features: (1) HOMA insulin sensitivity decreases steeply 5 years prior to T2D occurrence; (2) FG undergoes abrupt increase 2-3 years before T2D onset; (3) significant elevation of A1c is not observed until 1 year before the diagnosis of T2D (Supplemental Figure  1) [24–26]. The different trajectories rank HOMA insulin sensitivity (or insulin resistance, assessed by HOMA-IR) higher than FG and A1c as a potential marker for identifying earlier stage of prediabetes. Indeed, HOMA-IR and FI showed significant correlation with the risk index BMI (Figure 2). Moreover, insulin resistance was significantly associated with the changes in mitochondrial ND6 and D-loop DNA methylation, while the latter showed no correlation with A1c or FG (Figure 3). Based on quartiles of BMI, mitochondrial epigenetic traits underwent significant changes from healthy (20 < BMI < 24.9, Q1) to obese (30 < BMI < 34.9, Q2) status, which corresponds to the development of insulin resistance (Figure 4, Supplemental Figure  1). However, no significant change was observed in FG or A1c during the Q1 → Q2 transition (Supplemental Figure  5). These findings suggest that mitochondrial epigenetic changes represent an early-stage event in prediabetes progression and is associated with the development of insulin resistance.

The Prevent Diabetes STAT campaign calls for early identification and intervention to prevent the future development of T2D [53]. Thus, our ability to consistently identify prediabetic individuals as early as possible in the disease progression stages will be of utmost importance to address these challenges. In this study we examined the diagnosing power of FG and A1c by following the standards recommended by ADA [12]. FG indicated that 27.6% of individuals were prediabetic, 1.1% diabetic, and 71.3% healthy or normal. By A1c, however, 49.4% of the tested participants were considered prediabetic and 3.5% diabetic, leading to a diagnostic rate 1.8-fold and 3.2-fold higher than FG, respectively (Figure 1). In addition, neither FG nor A1c showed significant correlation with BMI, the primary risk index used for screening (Figure 2), which may account for the wide variation in diagnosis thus highlighting the urgent need for additional diagnosis markers [14, 15]. More importantly, mitochondrial epigenetic signature was significantly correlated with BMI and showed robust changes in early stage of prediabetes progression that corresponds to the development of insulin resistance (Figures 3 and 4). These results suggest that mitochondrial epigenetic marker may have greater potential than A1c or FG to achieve early diagnosis of prediabetes. Future studies seeking to determine the value of mitochondrial epigenetic signatures in accurately diagnosing prediabetes and assessing intervention effectiveness will be of critical importance to fulfill the mission of Prevent Diabetes STAT.

Taken together, effective prevention of T2D requires as-early-as-possible diagnosis of prediabetes for lifestyle intervention. We showed that the current diagnosis indices A1c and FG led to significant discordance, underscoring the urgent need of additional markers to achieve reliable diagnosis. Importantly, we found that the mitochondrial epigenetics signature (DNA methylation in ND6 and D-loop) characteristically signified the early stage of prediabetes when insulin resistance develops. By contrast, advanced stages of prediabetes progression lead to no significant changes in mitochondrial epigenetic signature. Thus, mitochondrial epigenetic changes represent an early-stage event in prediabetes progression, and further studies to systematically examining mitochondrial epigenetic signature in prediabetes diagnosis, particularly in larger-scale cohorts, will be of critical importance.

## Supplementary Material

The progression of prediabetes to T2D showed stage-dependent features in insulin sensitivity, fasting glucose (FG) and hemoglobin A1c. As the primary T2D risk index, body mass index (BMI) is significantly correlated with impaired insulin sensitivity but not with FG or A1c. Consistently, A1c shows marginal changes in all quartiles of BMI, and FG shows changes only in the very high-risk stages, i.e., Q3 (severely obese) and Q4 (morbidly obese), in comparison with healthy or low-risk stage (Q1). Although lipid profile was altered in overweight and obese individuals, no correlation was observed between lipids and mitochondrial epigenetic changes.

## Figures and Tables

**Table 1:** Demographic and metabolic characteristics of participants.

Characteristics	Lean (*n* = 12)	Overweight/obese (*n* = 75)
Sex (male/female)	3/9	22/53
Age (years)	25.7 ± 3.1	48.4 ± 1.5^*∗∗∗*^
BMI (kg/m^2^)	22.7 ± 0.6	37.1 ± 0.8^*∗∗∗*^
Fasting glucose (mg/dL)	83.4 ± 1.8	96.1 ± 1.5^*∗∗*^
Fasting insulin (*μ*IU/mL)	10.0 ± 1.5	24.6 ± 2.4^*∗*^
HOMA-IR	2.1 ± 0.3	6.0 ± 0.6^*∗*^
HbA1c (%)	5.5 ± 0.1	5.7 ± 0.1
HDL (mg/dL)	61.9 ± 4.2	54.7 ± 1.4
LDL (mg/dL)	90.8 ± 6.7	114.1 ± 3.5^*∗*^
LDL/HDL ratio	1.6 ± 0.2	2.2 ± 0.1^*∗*^
VLDL (mg/dL)	17.6 ± 2.2	28.4 ± 2.0^*∗*^
Total cholesterol (mg/dL)	170.1 ± 5.5	195.9 ± 3.9^*∗*^
Total cholesterol/HDL	2.9 ± 0.2	3.7 ± 0.1^*∗∗*^
Triglyceride (mg/dL)	88.1 ± 10.9	140.7 ± 10.1^*∗*^

Mean ± SE; ^*∗*^ *p* < 0.05; ^*∗∗*^ *p* < 0.01; ^*∗∗∗*^ *p* < 0.001.

## Abbreviations

A1c: Glycated hemoglobin A1c
BMI: Body mass index
D-loop: Displacement loop
FG: Fasting glucose
FI: Fasting insulin
HOMA: Homeostatic model assessment
HOMA-IR: HOMA insulin resistance index
IFG: Impaired fasting glucose
LINE-1: Long interspersed nucleotide element 1
LUMA: Luminometric methylation assay
ND6: Mitochondrial NADH dehydrogenase 6
T2D: Type 2 diabetes.
